# Assessing Grief in Cancer Care: A Systematic Review of Observational Studies Using Psychometric Instruments

**DOI:** 10.3390/healthcare13141722

**Published:** 2025-07-17

**Authors:** Rebecca Mattson, Margaret Henderson, Savitri Singh Carlson

**Affiliations:** 1 School of Nursing, San Diego State University, San Diego, CA 92182, USA; ssinghcarlson@sdsu.edu; 2 San Diego State University Library, San Diego State University, San Diego, CA 92182, USA; margaret.henderson@sdsu.edu

**Keywords:** cancer, grief, psychometric instruments, psychological distress, oncology, quality of life

## Abstract

**Background/Objectives**: Grief in cancer patients represents a multidimensional psychological response encompassing anticipatory, existential, and identity-related distress. While the recent literature has examined grief in caregivers, family members, and even healthcare professionals, the psychological grief experienced by patients themselves following a cancer diagnosis remains comparatively understudied and insufficiently characterized in empirical research. This systematic review aims to evaluate observational studies that used validated psychometric instruments to measure grief in adult cancer patients and to synthesize findings on the significance of grief in this population. **Methods**: Following PRISMA 2020 guidelines, a systematic search of PubMed, CINAHL, and PsycINFO was conducted to identify observational studies that employed validated tools to assess grief among adult cancer patients. The inclusion criteria required the use of psychometrically validated grief instruments and the collection of quantitative data. Fifteen studies met eligibility criteria and were included in the final analysis. **Results**: Grief symptoms were consistently present at moderate to high levels across diverse cancer types, care settings, and geographic regions. Preparatory Grief in Advanced Cancer (PGAC) scores often exceeded thresholds associated with clinical concern, with correlations observed between grief and psychological variables such as anxiety (r = 0.63), depression (r = 0.637), hopelessness (r = 0.63), and dignity (r = 0.654). Demographic factors (e.g., younger age, female gender) and illness perceptions (e.g., identity centrality, stigma) further intensified grief. Grief was a predominant psychological concern even when general distress measures failed to capture its presence. **Conclusions**: Future research is essential to identify an effective public health strategy for addressing grief through structured screening conducted in primary care and outpatient medical settings, coupled with accessible referral pathways to community-based support groups and coordinated follow-up services to facilitate grief management.

## 1. Introduction

Cancer is a leading cause of mortality worldwide and a significant public health challenge, with global incidence and deaths rising steadily, an estimated 20 million new cases and 9.7 million cancer-related deaths in 2022 [1]. Beyond the physical burden of disease, cancer imposes profound psychosocial impacts. A significant proportion of patients experience emotional distress during the cancer trajectory [2]. These psychosocial challenges include anxiety, depression, and existential distress, as well as grief, an often under-recognized reaction in patients coping with cancer-related losses [2]. Psycho-oncology, a relatively new interdisciplinary field bridging oncology and mental health, has emerged to address such issues and provide support for patients. Psycho-oncological interventions focus on helping patients cope with fear, grief, pain, and fatigue to improve quality of life, in both clinical care and broader population-level contexts.

Many people view a cancer diagnosis as a death sentence. This perception may intensify grief and complicate emotional adaptation [3]. Grief encompasses psychological responses to expected losses, including diminished health, disrupted identity, and altered future expectations [3]. The progression of cancer and the process of dying are often unpredictable and non-linear, compounding emotional distress and uncertainty.

Grief is an inherent and multifaceted component of the cancer experience, encompassing physical, emotional, psychological, and social dimensions [4]. Typically, grief in the face of death is considered a normal reaction; however, it must be expressed and acknowledged [5]. A cancer diagnosis often precipitates a profound sense of loss, accompanied by fears related to disease progression, mortality, and loss of control, thereby affecting not only patients but also their families, caregivers, and healthcare providers [3]. While there is a depth of research on caregiver, family, and provider grief, there remains a gap in studying grief in the actual patient population.

The previous literature focuses on grief in families, caregivers, and health care professionals; a limited amount of the literature evaluates the concept of grief in patients with a cancer diagnosis. Family and caregivers of cancer patients experience what is called anticipatory grief, while the patient is still alive, and bereavement-related grief after the patient’s death. Research studies indicate that grief is prevalent among caregivers, with up to 25% of them experiencing anticipatory grief, a condition that is linked to caregiver burden, family resilience, and coping strategies [6]. Grief in caregivers is seen as early as the time of diagnosis and can show up as worry, tearfulness, sleep disturbance, guilt, and loneliness. This emotional impact has a profound effect on the quality of life and health of caregivers, which is why a substantial body of knowledge addressing this topic has emerged [4,7,8,9]. Caregivers’ grief often continues after the loss of the patient; this can be described as Prolonged Grief Disorder (PGD). Findings show that 11.3% of caregivers meet this diagnosis 11 months after the loss [10]. A systematic review in 2020 confirmed that high pre-loss grief in caregivers is associated with a high rate of prolonged grief and depression after someone passes [11]. Guidelines now call for assessing caregivers’ needs and offering grief counseling both before and after a patient’s death [10]. Beyond primary caregivers, family members like spouses, children, and siblings experience multiple losses as the once healthy person they knew now has a different role before any death [10,11,12,13]. Grief is also acknowledged among healthcare professionals, including medical doctors and oncology nurses, who provide care for cancer patients and establish bonds with them [14,15]. Research indicates that oncology fellowship training, peer support groups for staff after a patient’s death, and institutional policies that acknowledge and support provider grief help maintain emotional well-being and support those who are providing oncology care, thereby reducing compassion fatigue [14]. Cancer patients grieve the loss of health, independence and changes in their body, role, life, and plans [16]. This sense of loss can contribute to what is called disenfranchised grief in patients, which describes grief that is not openly acknowledged by society and may not involve death [16,17,18]. For those with a terminal diagnosis, patients may have preparatory grief, which is grief in anticipation of their death and sadness over events that they will miss [19]. Fear of death and the anticipation of the loss of life are emotions that those with a cancer diagnosis, especially a terminal one, experience. Those who have high levels of preparatory grief may even experience greater emotional distress, such as depression [16,19]. Although grief responses are highly individualized and shaped by cultural norms, personal beliefs, and spiritual values, the experience of grief is nearly universal and characterized by emotional and existential distress [18].

Grief experienced by cancer patients is closely tied to their perceptions of the illness, especially concerning its effects on identity and social engagement. Significant factors influencing grief include the importance of identity, the stigma linked to the diagnosis, and the level of hope the patient feels. For instance, cancer can change an individual’s self-perception, resulting in feelings of isolation or shame due to social stigma, while nurturing hope can ease grief and promote resilience. Patients themselves may need grief support, just as their families do, even while they continue to live with cancer. Despite its significance; grief remains under-assessed in routine oncology care. Tools like the Distress Thermometer are widely used but may not adequately capture the nuances of anticipatory grief. Instruments such as the Preparatory Grief in Advanced Cancer (PGAC) scale have been developed to provide more targeted assessment; however, their integration into clinical workflows is limited [20]. In the absence of standardized protocols for grief screening, clinicians may miss opportunities to identify distress and provide timely psychosocial support. Unaddressed grief can hinder access to appropriate care and negatively affect the emotional well-being of patients and their support networks. The objective of this work is to evaluate existing screening practices for grief among cancer patients, identify which psychometric tools are in use, and assess their applicability in capturing the multidimensional experience of grief. Through a synthesis of observational studies, this review examines the utility of grief assessment tools and advocates for their systematic inclusion in comprehensive cancer care.

## 2. Materials and Methods

### 2.1. Study Design and Protocol

This systematic review adhered to the Preferred Reporting Items for Systematic Reviews and Meta-Analyses (PRISMA) 2020 guidelines (https://doi.org/10.1136/bmj.n71, accessed on 11 May 2025). The review focused on observational studies that assessed grief in adult cancer patients using validated psychometric instruments. The protocol was registered at OSF, https://osf.io/eru7w (accessed on 11 May 2025).

### 2.2. Eligibility Criteria

Studies were included if they (i) employed an observational design; (ii) involved adult cancer patients (≥18 years); and (iii) used a validated psychometric instrument to assess grief.

The exclusion criteria were (i) studies focusing solely on bereavement in caregivers or family members; (ii) studies without a grief-specific psychometric tool; (iii) non-English-language publications; and (iv) qualitative, longitudinal, or interventional study designs.

### 2.3. Search Strategy and Information Sources

A comprehensive literature search was conducted in PubMed, CINAHL, and PsycINFO (via EBSCO) from the inception of the databases (1966, 1937, 1800s) to 31 May 2024. Search terms included combinations of keywords and controlled vocabulary (e.g., “cancer,” “grief,” “psychometric”) and were tailored to each database. Full search strategies are provided in Appendix A. The search was limited to peer-reviewed articles in English involving human participants.

### 2.4. Study Selection

**Figure 1:**
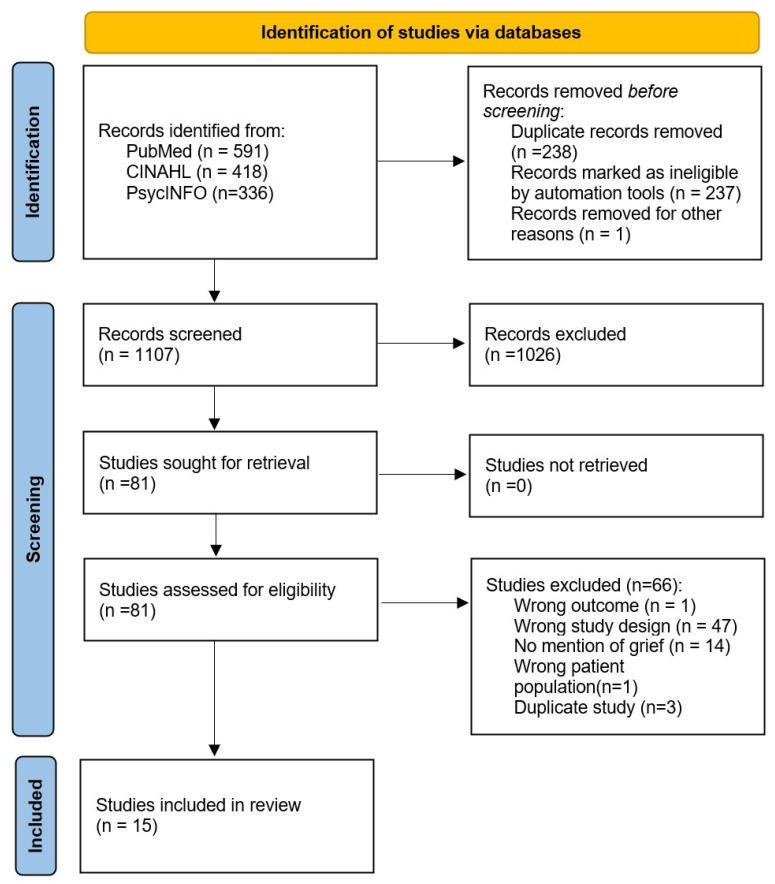
PRISMA flow diagram for study selection.

All retrieved references (n = 1345) were imported into Covidence systematic review software (Veritas Health Innovation, Melbourne, Australia) for screening. A total of 237 duplicates were removed automatically, and one additional duplicate was excluded manually. The remaining 1107 studies underwent title and abstract screening by two independent reviewers. Of these, 1026 were excluded due to irrelevance. In total, 89 full-text articles were assessed for eligibility, resulting in the exclusion of 71 studies for reasons including the use of non-validated psychometric instruments, a focus on caregiver grief, or an ineligible design. In three studies, we found that authors used the same study population and dataset for multiple analyses and papers; when this occurred, only the original (first paper) was included in the analysis. Fifteen studies met all the inclusion criteria, were not duplicate studies, and were retained for analysis. The PRISMA flow diagram is presented in Figure 1.

### 2.5. Data Extraction and Synthesis

Data were extracted independently by two reviewers using a standardized form. The extracted items included author(s), year of publication, country, study setting, sample characteristics, cancer type, grief assessment instrument, and outcomes related to prevalence, mediators, or care impact. Discrepancies were resolved through discussion and consensus.

### 2.6. Quality Appraisal

Although formal risk of bias scoring was not conducted due to the descriptive aim of the review, all included studies were screened for methodological clarity and adherence to reporting standards regarding instrument validity and study design (See Table 1).

### 2.7. Generative AI Statement

Generative artificial intelligence (GenAI) tools were utilized to assist in the manuscript’s conceptual organization and linguistic refinement. This included support in restructuring content for logical flow, academic tone, and consistency with MDPI formatting requirements. The authors reviewed and substantively edited the final content to ensure accuracy and scholarly integrity.

## 3. Results

We analyzed 15 observational studies that evaluated grief among cancer patients with validated psychometric tools. The studies employed validated grief assessment instruments such as the State Hope Scale, the Preparatory Grief in Advanced Cancer Patients (PGAC) Scale, the Prolonged Grief Disorder Scale (PG-12 and PG-13), and the Basic Documentation for Psycho-Oncology (PO-Bado). Sample sizes varied among the studies, representing a range of demographics, cancer types, disease stages, and psychosocial factors.

### 3.1. Demographics

The demographic characteristics of the study populations across the 15 included studies were examined.

Studies employing the Preparatory Grief in Advanced Cancer Patients (PGAC) scale were primarily conducted in Greece, across both inpatient and outpatient settings for palliative care. Kostopoulou et al. [21] studied 120 patients (55% female) in Athens, with a mean age of 66.4 years. Cancer types included lung (26.7%), gastrointestinal (15%), and breast cancer (15%). Mystakidou et al. [22] investigated 220 patients (55.5% female, mean age 61.7), most of whom were married (73%) and had only completed primary school. Their sample had a high prevalence of metastatic disease (70.5%) and poor ECOG performance status (58.5%). Mystakidou et al. [23] also studied 94 patients (56.4% female, mean age 57.7), all with Stage IV cancer. Gastrointestinal (31.9%), urogenital (23.4%), and breast cancers (21.3%) were common, and 19.1% of participants had confirmed metastases. A similar 2008 study from the same group [24] examined 100 terminal cancer patients (53% female, mean age 63.2), reporting a wide range of cancer types and educational attainment; over half had primary education. Mystakidou et al. [25] studied another 94 patients (51% female, mean age 63.4), with 90.4% reporting a partner and an average of 9.66 years of education. Cancer diagnoses included urogenital (35.1%), gastrointestinal (18.1%), lung (18.1%), and breast (12.8%). In a subsequent study, Mystakidou et al. [26] evaluated 195 patients with Stage III–IV cancer (mean age 64.4), most of whom were married and had optimal ECOG performance. Urogenital cancer was most common (26.7%), and 85.6% had metastatic disease. Parpa et al. [27] assessed 120 advanced cancer patients (55% female, mean age 66.4) using PGAC, HADS-D, and the Patient Dignity Inventory (PDI), with cancer diagnoses similar to prior PGAC studies. Tsilika et al. [28] studied 94 patients (51% female, mean age 63.4) with Stage IV cancer, with 69.1% showing good performance status and 68.1% presenting with metastases. Most were receiving chemotherapy (70.2%) and opioids (68.8%). Vergo et al. [19] conducted the only U.S.-based PGAC study, involving 53 patients at a cancer center in Chicago. The sample was nearly evenly split by sex, and 55% of patients had gastrointestinal cancer. Despite having an incurable disease, 28% of patients did not perceive their condition as terminal.

Siedentopf et al. [29] utilized the Psycho-Oncological Base Documentation (PO-BADO) instrument to assess 333 German breast cancer patients undergoing surgery. The mean age was 59.9 years, with 21.9% under 50 and 38.1% over 64. Most participants (63.9%) were partnered. Employment status indicated that 50.9% were retired and 35.5% employed. Over half reported comorbidities (52.7%) and approximately 22% had prior psychological treatment. Nearly all participants (97.2%) had been diagnosed within the previous month.

Tacón et al. [30] used the Grief Diagnostic Instrument (GDI) in a study of 65 U.S. women recently diagnosed with breast cancer. The mean age was 45.4 years, with the sample being predominantly white (94%) and middle class (92%). Educational attainment was high, with 68% having education beyond high school. Most participants were Protestant (96%) and married (60.8%). Employment data showed that 52% worked part-time. Stage I (68%) and Stage II (32%) cancers were reported. A majority were receiving chemotherapy or radiation at the time of the study.

The Prolonged Grief Disorder Scale (PG-12) was used in three studies by Trevino and colleagues to assess grief among young adults with advanced cancer. The 2011 study [31] included 53 participants (66% female, mean age 33.9), 92.5% of whom were white. Nearly half were married, and 41.5% had dependent children. Most had incomes above USD 50,000, and the average educational level was 15.5 years. Breast cancer was the most common diagnosis (39.6%), with most patients diagnosed at Stage III or IV. The 2013 study [32] on social support included 71 participants (70.4% female, mean age 34.0), with similar socioeconomic and clinical profiles. Nearly half had metastatic disease, and educational attainment averaged 15.8 years. The third study [33] on patient-oncologist alliance included 95 participants (68.4% female, mean age 33.4). The cohort predominantly consisted of individuals who were white and possessed a high level of education, with more than 50% earning in excess of USD 50,000 annually. Breast cancer remained the most prevalent diagnosis, and over half of the sample had metastatic disease at the time of participation. In the study utilizing the PG-12, Gökler-Danışman [34] examined 250 cancer patients in Istanbul, Turkey. The sample consisted of 67.6% females and 32.4% males, with participants representing diverse educational backgrounds, including elementary (24.6%), high school (32.3%), and university-level (28.6%) education. The majority of participants were middle-income (78.2%), with lower- and higher-income individuals comprising 12.9% and 8.9%, respectively. Employment was low, with 74.4% unemployed and 8.0% having left work due to illness. Participants primarily resided in metropolitan areas (82.8%) and were diagnosed with a range of cancers, including breast (29.9%), lung (17.0%), and others such as lymphoma and gastric cancer.

**Table 1:** Summary of location, instrument, and demographic information from the included studies [19,21,22,23,24,25,26,27,28,29,30,31,32,33,34].

Study	Location	Psychometric Grief Instrument	Demographics
Gökler-Danışman, 2017 [34]	Turkey	PG-12	n = 250, 67.6% female, 32.4% male, mean age 55.8 ± 12.96
Kostopoulou, 2018 [21]	Greece	PGAC	n = 120, 55% female, 45% male. mean age 66.4 ± 13.64 (range 29–95)
Mystakidou, 2006 [22]	Greece	PGAC	n = 200, 55.5% female, 44.5% male, mean age 61.7 (range 31–87)
Mystakidou, 2008 [23]	Greece	PGAC	n = 94, 56.4% female, 43.6% male, mean age 57.65 ±14.0 (range: 24–85)
Mystakidou, 2008 [24]	Greece	PGAC	n = 100, 53% female, 47% male, mean age 63.17 ±15.3
Mystakidou, 2011 [25]	Greece	PGAC	n = 94, 51% female, 49% male, mean age 63.37 years ± 12.03, (range: 41–93)
Mystakidou, 2012 [26]	Greece	PGAC	n = 195, mean age of 64.41 ± 12.37, (range from 34–93)
Parpa, 2019 [27]	Greece	PGAC	n = 120, 55% female, 45% male, mean age 66.45 years ±13.64 (range 29–95).
Siedentopf, 2010 [29]	Germany	PO-Bado	n = 333
Tacón, 2011 [30]	Texas, USA	GDI	n = 65 women, mean age 45.4 (range 32–63)
Trevino, 2011 [31]	Massachusetts, USA	PG-12	n = 53, 66% female, 34% male, age range 20–40
Trevino, 2013 [32]	Massachusetts, USA	PG-12	n = 71, 70.42% female, 29.58% male, age range 20–40
Trevino, 2013 [33]	Massachusetts, USA	PG-13	n = 97, 68.4% female, 31.6% male, mean age 33.4 ± 5.51 (range 20–40)
Tsilika, 2009 [28]	Greece	PGAC	n = 94, 51% female, 49% male, mean age 63.37 ± 12.03 (range: 41–93)
Vergo, 2017 [19]	Multiple states, USA	PGAC	n = 53, 53% female, 47% male, mean age 63.37 years ± 12.03 (range: 41–93)

### 3.2. Grief Scales

Five psychometric instruments were utilized across the included studies to assess grief among individuals with cancer. These instruments varied in length, scoring range, and intended use context.

The Preparatory Grief in Advanced Cancer Patients (PGAC) scale is a 31-item, unidimensional tool developed to assess grief specifically related to the anticipatory loss experienced by individuals with terminal cancer. Items are rated on a 5-point Likert scale (0 = not at all to 4 = very much), yielding a total score range of 0 to 124. It assesses emotional reactions such as sadness, future loss orientation, role identity conflict, and existential distress.

The Psycho-Oncological Base Documentation (PO-Bado) is a semi-structured clinician-administered interview that includes both somatic and psychological symptom domains. The psychological domain comprises six items, one of which addresses explicitly grief-related symptoms—grouped with despondency and depressive affect—rated on a 5-point scale (0 = not at all to 4 = very much). PO-Bado is used primarily for screening psychosocial needs in hospitalized oncology patients.

The Grief Diagnostic Instrument (GDI) is a 16-item self-report measure designed to identify the intensity and phenomenology of grief, including affective, cognitive, and behavioral responses. Responses are scored on a 4-point scale (0–3), with higher scores indicating more severe grief. The GDI was used to evaluate intervention responsiveness in a sample of women with early-stage breast cancer [30].

The Prolonged Grief Disorder Scale-12 (PG-12) and Prolonged Grief Disorder Scale-13 (PG-13) are standardized instruments developed to assess symptoms of prolonged or complicated grief. The PG-12 includes 12 items covering grief-related separation distress, cognitive and emotional dysregulation, and social impairment. The PG-13 expands this to 13 items and includes a duration criterion. Items are scored on a 5-point scale (1 = not at all to 5 = several times a day), yielding a possible total score of 13 to 65. These instruments have been validated in life-threatening illness populations, and Trevino et al. employed them to quantify grief in young adults with advanced cancer receiving care at a tertiary cancer center.

#### 3.2.1. Grief Findings

Researchers have investigated grief in cancer populations using a range of psychometric tools, each varying in theoretical basis, structure, and sample population. A comprehensive overview of empirical studies examining grief in individuals with cancer is presented in Table 2, which summarizes the psychometric instruments used, key grief-related findings, associations with anxiety and depression, reported confounders, and internal reliability (Cronbach’s alpha) for the grief scale if referenced or reported.

The most used measure is the Preparatory Grief in Advanced Cancer Patients (PGAC) scale, which was applied in nine studies. PGAC demonstrated high internal consistency, with reported Cronbach’s alpha values of 0.838 in several studies [19,22,24,27], and a range from 0.823 to 0.864 in Mystakidou et al. [25] (Table 2).

PGAC mean scores across studies ranged from 26.7 [19] to 44.5 [22,24]. In a demographic analysis, Mystakidou et al. [22] found that grief scores were higher among female patients and significantly associated with anxiety (r = 0.818) and depression (r = 0.659). Mystakidou et al. [23] confirmed significant correlations between PGAC scores and anxiety (r = 0.629) and depression (r = 0.489), along with hopelessness and metastasis.

Mystakidou et al. [25] examined post-traumatic stress and found that PGAC scores were significantly related to intrusion, avoidance, and hyperarousal symptoms from the Impact of Event Scale-Revised (IES-R) (r = 0.433–0.579). In a complementary investigation, Mystakidou et al. [24] evaluated PGAC as a screening tool and identified optimal cutoff scores of 40+, with strong performance in detecting clinical anxiety (AUC = 0.968) and depression (AUC = 0.867), based on the Hospital Anxiety and Depression Scale (HADS).

Tsilika et al. [28] extended these findings by modeling PGAC predictors and identifying significant contributions from intrusion (B = 4.11), hyperarousal (B = 8.43), younger age (B = −0.18), and ECOG performance status (B = 4.84), accounting for 51.5% of PGAC score variance.

Kostopoulou et al. [21] found PGAC to be associated with anxiety (r = 0.711), depression (r = 0.573), and aspects of body image, self-esteem, and social support. Parpa et al. [27] also linked PGAC to a diminished sense of dignity (r = 0.654), with depression mediating the relationship between grief and dignity. In a U.S.-based study, Vergo et al. [19] reported a mean PGAC score of 26.9 and found that general distress (Distress Thermometer) was the predictor of preparatory grief in multivariate regression (β = 3.2, *p* = 0.001).

Outside the PGAC framework, other grief instruments were used in studies involving adolescents and young adults (AYA) with advanced cancer. The Prolonged Grief Disorder Scale—12 item (PG-12) was used in four studies and demonstrated strong internal consistency, with Cronbach’s alpha values ranging from 0.76 to 0.89.

In Trevino et al. [31], the mean PG-12 score was 23.80 (SD = 7.05), with grief significantly associated with perceived life disruption (r = 0.46, *p* < 0.01). Gökler-Danışman et al. [34] reported a mean score of 26.09 (SD = 9.46) and identified grief as being positively associated with negative illness perceptions, including identity centrality, stigma-induced discrimination, and lower hopefulness. The authors reported a Cronbach’s alpha of 0.89.

Two studies by Trevino et al. [32,33] further explored PG-12 scores. In the first, more severe grief was associated with a greater likelihood of considering stopping treatment (r = 0.28, *p* = 0.009), and grief was inversely associated with alliance strength (r = –0.28), suggesting a potential behavioral impact. In the second, alliance with the oncologist was associated with reduced grief and improved adherence, while adjusting for appraisal support and metastasis. Both studies reported Cronbach’s alpha of 0.76 for the PG-12.

Tacón [30] applied the Grief Diagnostic Instrument (GDI) in a pre-post mindfulness study among women with breast cancer. Grief scores significantly decreased from 20.12 to 17.72 (t = 3.56, *p* < 0.01) and anxiety (anxious preoccupation subscale) also declined (M = 22.32 to 17.38; t = 5.74, *p* < 0.001). Cronbach’s alpha for the GDI was not reported.

Siedentopf et al. [29] used the PO-Bado (Psycho-Oncological Basic Documentation) in a German breast cancer sample and reported grief/despondency/depression as the most prevalent psychological concern (M = 1.59, SD = 1.17), with anxiety scores showing age-related differences (*p* < 0.001). Although internal reliability was not reported, this screening instrument highlighted the frequency of grief-related distress among patients.

**Table 2:** Summary of data extraction, including grief scores, findings, and Cronbach’s Alpha. [19,21,22,23,24,25,27,28,29,30,31,32,33,34].

Study	Location	Article Title	Psychometric Grief Instrument	Finding 1	Comparison 1	Comparison 2	Confounders	Cronbach’s Alpha
Tacón, 2011 [30]	Lubbock, Texas, USA	Mindfulness: Existential, Loss, and Grief Factors in Women with Breast Cancer	GDI	Mean (Pre-Intervention): 20.12 (SD = 4.67) Mean (Post-Intervention): 17.72 (SD = 4.14) t = 3.56	Anxious Preoccupation (M = 17.38, SD = 3.20), t = 5.74, *p* < 0.001	Essential Wellbeing M = 32.17, SD = 8. 02), t = 4.63, *p* < 0.001	Gender, Cancer Type, Time	Not reported
Mystakidou et al., 2006 [22]	Athens, Greece	Demographic and Clinical Predictors of Preparatory Grief	PGAC	Mean = 44.5 (SD = (13.6))	Anxiety symptoms: r = 0.818, *p* < 0.0005 (HADS-A).	HADS-D: r = 0.659, *p* < 0.0005 (HADS-D).	Age, ECOG status, gender, opioids, other surgery, surgery	PGAC α = 0.0838
Mystakidou et al., 2008 [23]	Athens, Greece	Preparatory grief, psychological distress and hopelessness	PGAC	Mean = 42.49 (SD = (13.97))	HADS-A PGAC: r = 0.629, *p* < 0.0005	HADS-D: r = 0.489, *p* < 0.0005	Age, metastasis, gender, education, chemo-, radiotherapy, cancer type	PGAC α = 0.70
Mystakidou et al., 2008 [24]	Athens, Greece	Screening for Preparatory Grief in Advanced Cancer Patients	PGAC	Mean = 44.5 (SD = (13.6))	Anxiety AUC 0.968 SE 0.014 *p* < 0.0005	Depression AUC 0.867 SE 0.036 *p* < 0.0005	ECOG status, diagnosis, treatment, opioid use	PGAC α = 0.838 (0.823–0.864)
Mystakidou et al., 2011 [25]	Athens, Greece	The Mediation Effect of Anxiety Between PTSD and Grief	PGAC	Mean = 35.88 (SD = (10.42))	Anxiety (r = 0.527); PTSD symptoms (IES-R total) r = 0.433–0.579	IES-R- hyperarousal (1.65, 0.70)	Post-traumatic stress, ECOG Status, Cancer type, Treatment	PGAC (no alpha, reported as Spearman’srhocoefficient) HAD α = 0.887 Anxiety 0.703 Depression
Parpa et al., 2019 [27]	Greece	Depression as Mediator Between Grief and Dignity	PGAC	Mean = 27.58 (SD = (14.34))	Patient Dignity Inventory (PDI) score r = 0.637, *p* < 0.001	HADS-D r = 0.565, *p* < 0.001	Cancer stage, Gender	PGAC α = 0.0838
Vergo et al., 2017 [19]	Chicago, IL, USA	Assessing Preparatory Grief in Advanced Cancer Patients as an Independent Predictor of Distress in an American Population	PGAC	Mean PGAC = 26.7 (range = 12.0–41.4)	HADS-A Score Change %, CI 1.72 (0.99, 2.46) *p* < 0.0001	HADS-D Score Change %, CI 1.74 (0.98, 2.51).	Distress thermometer, HADS, satisfaction with QoL	PGAC α = 0.0838
Kostopoulou et al., 2018 [21]	Athens, Greece	Advanced Cancer Patients’ Perceptions of Dignity: The Impact of Psychologically Distressing Symptoms and Preparatory Grief	PGAC	Mean = 27.58 (SD = (14.34))	PGAC scores associated with HADS-Anxiety range r = 0.711 to 0.330 across subscales.	HADS-D r = 0.573 to 0.314 across subscales	Age, gender, education, ECOG, stage, chemo-, hormonotherapy	PGAC α = 0.838
Tsilika et al., 2009 [28]	Athens, Greece	The Influence of Cancer Impact on Patients’ Preparatory Grief	PGAC	Mean PGAC = 35.88, SD = (10.42)	PTSD subscales: Avoidance r = 0.537, Intrusion r = 0.607, Hyperarousal r = 0.645 *p* < 0.0005	IES-R Total r = 0.70, *p* < 0.0005	Age, ECOG, intrusion, hyperarousal	PGAC α = 0.838
Siedentopf et al., 2010 [29]	Germany	Experiences with a specific screening instrument to identify psychosocial support needs in breast cancer patients	PO-Bado	Grief/despondency/depression: M = 1.59, SD = (1.17)	Anxiety/worries/tension: M = 1.57, SD = (1.29); *p* < 0.001	Age-grouped means (<50 y) grief scores (M = 1.90) than older (>64 y; M = 1.38), *p* = 0.009	Age, psychiatric history, tumor size, type of surgery	Not reported
Gökler-Danışman et al., 2017 [34]	Istanbul, Turkey	Experience of grief by patients with cancer in relation to perceptions of illness	PG-12	Mean grief score = 26.09 (SD = (9.46))	Illness perception (r = 22, *p* < 0.01)	Negative experience discrimination (r = 0.22, *p* < 0.01),hopeful ness (r = 0.25, *p* < 0.01), identity Centrality (r = 0.28, *p* < 0.01),	Gender, Education Level, Economic Level, Employment Status, Residence, Type of Cancer	PG-12 α = 0.89
Trevino et al., 2011 [31]	Dana-Farber Cancer Institute, Boston, MA	Grief and Life Disruption in Young Adults with Advanced Cancer	PG-12	Mean PG-12 = 23.80, SD = (7.05)	Life Disruption F (2, 49) = 13.06, *p* < 0.001	Education 15.49 (2.30), Spearmans rho −16; −0.40 Performance Status 77.55 (11.420, −0.39, 0.61)	Gender, marital status, dependents, income, metastatic status, trial status	PG-12 α = 0.76
Trevino et al., 2013 [32]	USA	Correlates of social support in young adults with advanced cancer	PG-12	Mean = 24.34, SD = (7.15)	QoL (r = –0.67, *p* < 0.1)	QoL (r = –0.53, *p* < 0.001)	Appraisal support, metastasis	PG-12 α = 0.76
Trevino et al. 2013 [33]	USA	Patient-oncologist alliance, psychosocial well-being, and treatment adherence among young adults with advanced cancer	PG-12	Grief and stopping treatment: r = 0.28, *p* = 0.009	Appraisal support r = 0.32, *p* = 0.002	Alliance r = −0.28, *p* = 0.01	Metastatic disease	PG-12 α = 0.77

Abbreviations: PGAC (Preparatory Grief in Advanced Cancer Patients Scale), PG-12 (Prolonged Grief Disorder Scale—12-item version), GDI (Grief Diagnostic Instrument), PO-Bado (Psycho-Oncological Basic Documentation Interview), HADS-A (Hospital Anxiety and Depression Scale—Anxiety subscale), HADS-D (Hospital Anxiety and Depression Scale—Depression subscale), IES-R (Impact of Event Scale—Revised), ECOG (Eastern Cooperative Oncology Group Performance Status), QoL (Quality of Life), IES-R-Grs (post-traumatic stress symptoms), PDI (Patient Dignity Inventory), AUC (Area Under the Curve), α (Cronbach’s alpha).

#### 3.2.2. Quality Assessment

All included studies were assessed for methodological rigor by evaluating psychometric properties relevant to instrument use, including validity, reliability, and potential sources of bias (Table 3). This quality appraisal was conducted independently by two reviewers and finalized through consensus in Convidence, using a custom risk of bias comparison tool. The tool incorporated five domains: (1) other sources of bias, (2) validity for all outcomes, (3) reliability for all outcomes, (4) sensitivity and specificity, and (5) measurement error. Each domain was rated as low, unclear, or high risk of bias, and textual annotations from each study supported judgments. This appraisal did not determine inclusion or exclusion; rather, it provided context for interpreting the strength and generalizability of psychometric evidence across studies.

**Table 3:** Risk of bias comparison [19,21,22,23,24,25,27,28,29,30,31,32,33,34].

Study	Validity	Reliability	Sensitivity and Specificity	Measurement Error	Other Sources of Bias
Gökler-Danışman, 2017 [34]	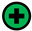	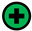	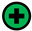	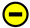	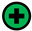
Kostopoulou, 2018 [21]	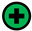	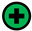	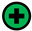	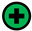	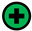
Mystakidou, 2006 [22]	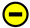	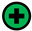	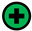	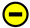	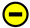
Mystakidou, 2008 [23]	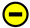	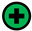	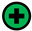	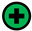	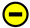
Mystakidou, 2008 [24]	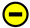	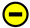	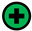	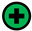	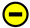
Mystakidou, 2011 [25]	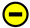	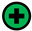	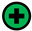	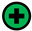	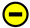
Mystakidou, 2012 [26]	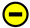	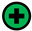	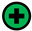	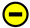	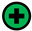
Parpa, 2019 [27]	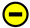	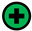	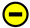	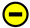	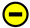
Siedentopf, 2010 [29]	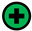	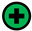	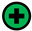	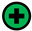	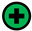
Tacón, 2011 [30]	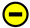	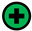	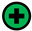	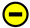	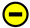
Trevino, 2011 [31]	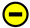	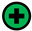	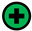	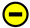	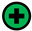
Trevino, 2013 [32]	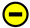	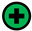	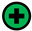	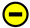	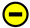
Trevino, 2013 [33]	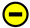	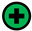	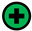	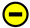	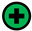
Tsilika, 2009 [28]	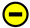	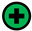	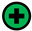	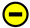	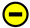
Vergo, 2017 [19]	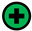	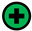	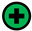	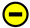	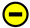

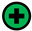
 Low Risk of Bias; 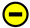 Unclear Risk of Bias

## 4. Discussion

Grief in patients with advanced cancer has historically received less attention than grief among caregivers or bereaved relatives. However, the recent psycho-oncology literature and clinical investigations emphasize the need to address the psychological, emotional, social, and behavioral dimensions of the cancer experience [35]. Preparatory or anticipatory grief in cancer patients has been shown to have higher levels of distress, such as depression, and warrants clinical support [6,19].

In this review of 15 observational studies, grief was consistently identified as a significant psychological burden among adult patients undergoing active cancer treatment or receiving palliative care. Instruments such as the PGAC scale, used in nine Greek cohorts led by Mystakidou et al. and one U.S. cohort, yielded mean scores ranging from 26.7 to 44.5, suggesting moderate to high levels of grief that often exceeded clinical thresholds of concern. Similarly, application of the PG-12 and PG-13 Prolonged Grief Disorder scales among young adults with advanced cancer demonstrated clinically significant mean scores of 23.8–24.3, reinforcing that such grief is not limited to caregivers or post-bereavement cohorts.

### 4.1. Grief Related to Emotional Distress

Despite its prominence, grief in patients remains under-assessed in routine oncology. Most screening protocols prioritize depression, anxiety, or distress without incorporating grief-specific instruments. Psychosocial care pathways rarely include structured grief interventions for patients, reserving such efforts for bereaved families or caregivers. This disconnect suggests a gap between empirical evidence and clinical practice. Instruments like the PGAC and PGD scales provide validated mechanisms for identifying and quantifying grief; however, their integration into standard oncology assessments remains limited. Addressing this gap is essential because intense grief can influence patients’ decisions about treatment, their involvement in palliative care, and their existential well-being. Its inclusion in psycho-oncological care paradigms, as an assessable and treatable construct, represents a necessary evolution in comprehensive cancer care.

### 4.2. Limitations

Most studies employed cross-sectional designs, which limited the ability to make causal inferences. Sample sizes were modest (n range 53–333) and often drawn from single centers, raising concerns about generalizability. Several Mystakidou et al. publications used potentially overlapping patient cohorts, making independent replication within Greece unclear. Cultural factors may influence grief expression and instrument performance; however, few studies have compared these across diverse populations.

### 4.3. Future Research

To advance grief care in oncology, future research should prioritize the integration of validated psychometric instruments within both quantitative and qualitative study designs. These instruments are needed for the reliable assessment of grief symptoms, facilitating early identification of patients at risk for prolonged or complicated grief. Their inclusion in randomized controlled trials and longitudinal cohort studies enables the systematic evaluation of structured grief screening protocols, with measurable outcomes such as psychological distress, quality of life, and referral rates to psychosocial services.

Incorporating psychometric measures into qualitative research can also enhance methodological rigor and interpretive depth. These instruments can inform patient care, provide a clinical baseline for exploring personal experiences, and anchor thematic analysis within established constructs. A mixed-methods approach may enhance the translational value of the findings, particularly when aligned with implementation science frameworks that assess feasibility, fidelity, and scalability across various healthcare settings. The strategic use of psychometric instruments thus offers a critical foundation for the development, evaluation, and dissemination of evidence-based grief care interventions in cancer populations.

## 5. Conclusions

This systematic review enhances the global understanding of grief in cancer patients by synthesizing evidence from 15 studies that explore grief as a distinct and clinically significant experience within the context of cancer. The findings advance the objectives of psycho-oncology by emphasizing the importance of directly assessing grief in patients as part of comprehensive cancer care. These studies demonstrate that grief can manifest across the illness trajectory—from diagnosis through advanced disease—and may have substantial implications for patients’ psychological and emotional well-being.

Despite this, grief in patients is frequently minimized or excluded from primary clinical focus. Much of the literature has concentrated on the grief experiences of caregivers, family members, or healthcare providers, with insufficient attention given to the patients themselves. This imbalance contributes to an enduring gap in care, where the emotional burden experienced by patients remains under-recognized and inadequately addressed.

Furthermore, most studies in this review employed observational designs, limiting causal inference and the ability to evaluate the effectiveness of grief-specific interventions. There is a clear need for more rigorous evidence through prospective cohort studies, randomized controlled trials, and qualitative research that centers on the lived experiences of patients. Expanding the methodological approaches in future research will be essential for developing targeted, patient-centered interventions that enhance the emotional and psychosocial dimensions of cancer care.

## Data Availability

No new data were generated. All data analyzed during this study are included in the published articles cited in the review. Extracted datasets are available from the corresponding author on reasonable request.

## Abbreviations

The following abbreviations are used in this manuscript:

PGAC	Preparatory Grief in Advanced Cancer Patients
G-HADS	Greek Hospital Anxiety and Depression Scale
G-SAHD	Greek Schedule for Attitudes toward Hastened Death
GDI	Grief Diagnostic Instrument
HADS	Hospital Anxiety and Depression Scale
HADS-D	Hospital Anxiety and Depression Scale—Depression Subscale
IES-R-Gr	Impact Events Scale Revised (Greek)
PDI	Patient Dignity Inventory
PG	Prolonged Grief Disorder Scale
DOAJ	Directory of open access journals
PO-Bado	Psychological-Oncological Base Documentation
QoL	Quality of Life

## Appendix A. Search Strategies Used in PubMed, CINAHL, and PsycInfo

PubMed

“cancer patient” [All Fields] OR “cancer patients” OR (“Patients” [Mesh] AND (“Neoplasms” [Mesh] OR cancer))

AND

(“Attitude to Death” [Mesh] OR “Grief” [All Fields] OR “mourn” [All Fields] OR “mourning” [All Fields] OR “Grief” [MeSH])

AND

Filters: Adolescent: 13–18 years, Adult: 19+ years

CINAHL

(MH “Neoplasms+” AND MH “Patients+”) OR MH “Cancer Patients”

AND

(MH “Grief+”) OR (grief OR mourn OR mourning) OR (MH “Attitude to Death”)

AND

Adult limiter

Psycinfo

((DE “Neoplasms” OR DE “Benign Neoplasms” OR DE “Breast Neoplasms” OR DE “Childhood Neoplasms” OR DE “Digestive System Neoplasms” OR DE “Endocrine Neoplasms” OR DE “Leukemias” OR DE “Lung Neoplasms” OR DE “Metastasis” OR DE “Nervous System Neoplasms” OR DE “Skin Neoplasms” OR DE “Terminal Cancer”) AND patient*)

AND

DE “Grief” OR grief OR mourn OR mourning

AND

Adult limiter
